# The relationship between obsessive-compulsive personality disorder traits, obsessive-compulsive disorder and excessive exercise in patients with anorexia nervosa: a systematic review

**DOI:** 10.1186/2050-2974-1-16

**Published:** 2013-05-02

**Authors:** Sarah Young, Paul Rhodes, Stephen Touyz, Phillipa Hay

**Affiliations:** 1 Clinical Psychology Unit, Mackie Building K01, School of Psychology, University of Sydney, Sydney, NSW, 2006, Australia; 2 School of Medicine, University of Western Sydney, Locked Bag 1797, Penrith, NSW, 2751, Australia; 3 School of Medicine, James Cook University, Townsville, North Queensland, Australia

**Keywords:** Anorexia nervosa, Exercise, Obsessive-compulsive disorder, Obsessive-compulsive personality, Excessive

## Abstract

**Objective:**

Obsessive-compulsive personality disorder (OCPD) traits and obsessive-compulsive disorder (OCD) are commonly associated with patients with Anorexia Nervosa (AN). The aim of this review was to systematically search the literature to examine whether OCPD and OCD are positively associated with excessive exercise in patients with AN.

**Method:**

A systematic electronic search of the literature (using PsycInfo, Medline and Web of Knowledge) was undertaken to identify relevant publications until May 2012.

**Results:**

A total of ten studies met criteria for inclusion in the review. The design of the studies varied from cross-sectional to retrospective and quasi-experimental. Seven out of the ten studies reviewed demonstrated a positive relationship between OCPD and/or OCD in AN patients who exercise excessively, whilst three studies found a lack of relationship, or a negative relationship, between these constructs.

**Conclusion:**

There is evidence from the literature to suggest that there is a positive relationship between OCPD and excessive exercise in patients with AN. However, the relationship between OCD and excessive exercise is less clear and further research is required to qualify the strength of such relationships. Future research should utilise the most comprehensive and reliable clinical assessment tools, and address prognostic factors, treatment factors and specific interventions for patients with OCPD and/or OCD and excessive exercise.

## Review

Anorexia Nervosa (AN) is recognised as one of the most serious chronic mental illnesses, with significant physical and psychosocial consequences [1]. In order to reduce burden of illness, increased understanding of developmental and maintaining factors of AN is required. The relationship between obsessionality and AN has been observed for a number of decades. However, research is yet to determine the distinct nature of this relationship and the putative moderating effect of excessive exercise [2].

Rothenberg [3] proposed that eating disorders are a “variant” of Obsessive-Compulsive Disorder (OCD), evidenced by high comorbidity between OCD and AN [4-6] and reporting of obsessional symptoms in AN patients [7]. AN patients follow strict food and exercise routines, and commonly present with obsessions of contamination and symmetry as well as compulsions of checking and counting [8-10]. There is also some neuro-chemical evidence for the relationship, with altered serotonergic function (5-HT) apparent in OCD and AN [11]. Although Obsessive-Compulsive Personality Disorder (OCPD) and OCD are recognised as distinct clinical syndromes, research has shown they significantly overlap in the risk profile for AN [12,13]. Yet, in their genetic studies, Lilenfeld, Kaye and colleagues demonstrated that while there was no shared causative factor for OCD and eating disorders within families, OCPD traits were demonstrated to be a specific familial risk factor for anorexia nervosa [14]. AN patients demonstrate personality traits which are highly concordant with OCPD- perfectionism, rigidity, higher impulse control and emotional restraint [15,16]. However, causal inferences are difficult to determine with obsessionality compounded by starvation effects [17]. AN patients show minimal changes in obsessional personality characteristics following weight restoration [18], suggesting that such pre-morbid personality traits play a role in the pathogenesis of AN [2].

Excessive exercise plays a detrimental role in the pathogenesis and maintenance of AN [19-21], and features in up to 80% of patients with AN [22]. The inverse relationship between reduced dietary intake and increased physical activity or hyperactivity- referred to as “activity anorexia” [23] was proposed as a bio-behavioural model of AN [21]. The presence of feeling “guilty” if an exercise session is missed is central to an eating disorder, signifying the obligatory nature of the behaviour [24] (also see [25,26] for models of the relationship between exercise dependence and eating pathology). Excessive exercise in AN has been associated with detrimental factors including: higher energy requirements for re-feeding/weight-gain [27]; elevated psychopathology [28]; and higher rates of relapse after recovery [29]. Based on the prognostic characteristics of excessive exercise, research has aimed to identify personality and psychological variables associated with this construct. AN patients who exercise excessively report higher levels of depression, anxiety and perfectionism [30,31], but potential relationships between excessive exercise and other psychological variables are thus far unclear.

### Rationale for the current review

The reviewed research suggests that AN is associated with increased OCD symptomatology and a higher prevalence of OCPD traits. Research is warranted to determine personality and psychological variables for excessive exercise, in particular those that may be remedial to interventions [32]. The aims are to critically examine evidence as to whether OCPD traits and/or OCD are associated with excessive exercise in AN, and to determine the nature of such relationships between these constructs in patients with AN.

## Method

### Search strategy

The search strategy was designed to identify all studies of patients with AN, in which OCPD or its traits, or OCD and its features were formally assessed, and in which excessive exercise was formally measured through clinical interview or clinical judgement.

The following databases were systematically searched from April-beginning of May 2012: PsycINFO (1806-present), Medline (1950-present) and Web of Knowledge (1864-present). Reference lists from relevant articles were also manually searched for additional studies. The following search terms were used: (anorexia* OR anore* OR eating disorder*) AND (exercise* OR excessive exercise* OR exercise abuse* OR over-exercise* OR compulsive exercise* OR exercise dependen* OR physical activit*) AND (personalit* OR obsessiv* OR obsessive compulsiv* OR compulsiv* OR OCD). Peer-reviewed research articles that focused on the relationship between exercise and obsessive compulsive disorder and/or obsessive compulsive personality traits in patients with anorexia nervosa were included. A total of 443 papers were retrieved from the electronic search. The titles and abstracts were screened to assess the suitability of papers. A second reviewer also screened a proportion of the titles and abstract to reduce selection bias. 79 papers were excluded from their title, and 302 papers were excluded from their abstract. The full text of 62 papers was read, and 54 were excluded. The reference lists of the final full text papers were searched manually, and a further two articles were retrieved. The second reviewer also read full texts of papers meeting the inclusion criteria, and there were no discrepancies in the inclusion of articles, thus a total of 10 studies were included in the review.

### Selection of studies

**Figure 1:**
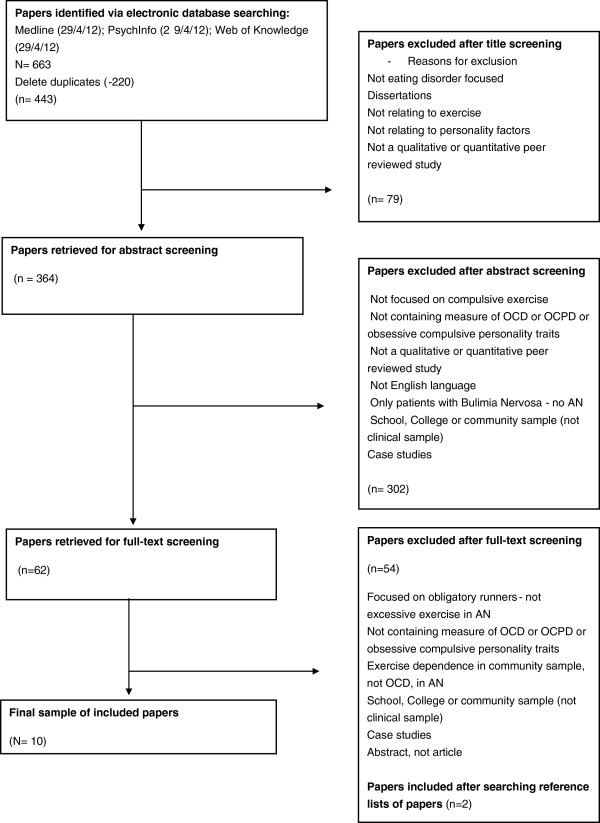
Flow chart of article retrieval process.

A detailed map of the search strategy can be seen in Figure 1. Papers were selected if: a) study was written (or available) in English; b) participants fulfilled standard DSM-IV-TR, DSM-IV, DSM-III or ICD-10 current or lifetime diagnosis criteria for AN; c) participants were assessed as having excessive exercise as a feature of their eating disorder; d) participants were assessed for OCPD traits or OCD symptoms/features using established scales/interview methods. Papers had to be published in peer reviewed journals, thus abstracts and dissertations were not included. There were no restrictions made on publication year, age or gender of participants, although the literature’s focus on females was aligned with current and historical low prevalence rates of AN in males. No restrictions were placed on participant’s Body Mass Index (BMI), the chronicity of illness in the sample, or the type of treatment received. Studies were excluded if the sample did not contain a group of patients with AN (if sample only included patients with Bulimia Nervosa or Eating Disorder Not Otherwise Specified). Studies were also excluded if they did not include a clinical sample: for example, only community samples, samples from university population or athlete samples without a sample of patients with diagnosed eating disorders.

### Quality assessment

The final retrieved articles underwent quality assessment utilising an amended version of the original Quality Index by Downs and Black [33]. The Quality Index is a reliable tool for measuring the methodological quality of epidemiological and health research [33]. This index had been amended by Ferro and Speechley [34] for their systematic review in the health science field. For their review, they excluded the assessment of quality items addressing characteristics of intervention studies, such as blinding, randomisation, withdrawals and drop-outs and integrity of intervention. The amended version resulted in 15 items (see Table 1). Each checklist item was scored 0 (No or Unable to Determine), or 1 (Yes). The maximum score was 15. A higher score indicated greater methodological rigour.

**Table 1:** Quality index of included studies (Ferro &Speechley, 2009, amended from Downs & Black, 1998)

	***Hypothesis clearly described***	***Main outcomes clearly described***	***Characteristics of patients described***	***Main findings clearly described***	***Estimates of random variability***	***Actual probability values used***	***Response rate clearly described***	***Patients -represent population***	***Patients prepared- represent population***	***Staff, place and facilities***	***Data dredging***	***Statistical tests appropriate***	***Outcome measures valid/reliable***	***Adjustment for confounding***	***Sample size or power calculation***	***Total***
Anderluh et al. (2009) [35]	1	1	1	1	0	1	0	0	0	0	1	1	1	1	0	9
Bewell-Weiss & Carter (2010) [36]	1	1	1	1	1	1	0	0	0	1	1	1	1	1	0	11
Davis & Claridge (1998) [37]	1	1	1	1	1	1	0	0	0	1	1	1	1	1	0	11
Davis & Kaptein (2006) [38]	1	1	1	1	1	1	0	0	0	1	1	1	1	1	0	11
Davis et al. (1998) [2]	1	1	1	1	1	1	0	0	0	1	1	1	1	1	0	11
Davis et al. (1995) [39]	1	1	1	1	1	1	0	1	0	1	1	1	1	1	0	12
Holtkamp et al. (2004) [40]	1	1	1	1	1	1	0	0	0	1	1	1	1	1	0	11
Naylor et al. (2011) [41]	1	1	1	1	1	1	1	0	0	1	1	1	1	1	0	12
Penas-Lledo et al. (2002) [31]	1	1	1	1	1	0	0	1	0	1	1	1	1	1	0	11
Shroff et al. (2006) [42]	1	1	1	1	1	1	0	1	0	1	1	1	1	1	1	13

## Results

### Description of studies

A total of ten studies were reviewed: four studies utilised AN participants who were receiving inpatient treatment; three studies used inpatients and outpatients; one study used outpatients only, whilst another study stated that they recruited from four eating disorder services, but did not specify the settings. The final study included patients with AN who were from the multisite international Price Foundation Study of AN, BN and AN Trios studies, and their affected relatives who met lifetime diagnosis of AN (see Tables 2 and 3 for a summary of the studies). Studies were conducted in a number of countries, including USA, Canada, Germany and Spain. Two other studies were conducted across more than one treatment site, such as in Slovenia and the UK. The majority of studies employed cross-sectional designs. One paper utilised a prospective design [38] and two used a retrospective design [31,35]. The final study was quasi-experimental [2].

**Table 2:** Characteristics, outcome measures and results of included studies

***Authors (Year) (Country)***	***Study design***	***Sample size/characteristic***	***Focus of study***	***Study schedule***
**Anderluh et al. (2009)**[35]**(Slovenia/UK)**	Cross-sectional study using clinical sample and retrospective reports	N = 97 female patients. ANR = 35, ANBP = 32, BN = 30. Mean current BMI for AN across subtypes = 16.7. Mean age of AN patients = 27.75 years.	Aimed to define an eating disorders (ED) phenotype by retrospectively assessing lifetime ED symptoms to examine a lifetime pattern of illness.	Initial screening diagnosis by experienced clinician in inpatient service. Participants interviewed by trained researcher. Demographic information collected, weight and height measured.
**Bewell-Weiss & Carter (2010) (Canada)**[36]	Cross-sectional design using clinical sample	N = 153 (148 female) first-admission inpatients with AN. Mean age = 26.0 years. Mean BMI = 15.0 kg/m^2^.	Aimed to amalgamate findings into a comprehensive regression model of predictors of excessive exercise in patients with AN.	All patients diagnosed by experienced clinician, with EDE. Exercise behaviour classified as excessive (34% of total N)-endorsed obligatory exercise, at least one hour daily, six days per week, for >1 month.
**Davis & Claridge (1998) (Canada/UK)**[37]	Cross-sectional design using clinical sample	83 female patients, AN = 34, BN = 49, Mean age = 28.1 years. Only patients without a history of another ED classification were included in the study.	Aimed to determine whether patients with eating disorders display addictive and OC personality characteristics relevant to weight preoccupation and excessive exercising.	Participants completed questionnaires and interview was conducted at time of admission to program.
**Davis & Kaptein (2006) (Canada)**[38]	Prospective design using clinical sample	50 inpatients-ANR. Mean age = 25.4 years. Mean BMI at admission = 14.05. BMI at discharge =19.6.	Aimed to determine whether AN patients represented a phenotype linked with OCD.	Completed questionnaires within first week of admission. Exercise interview conducted shortly after. Follow-up questionnaires were completed as soon as they attained target weight, determined by clinical team.
**Davis et al. (1998) (Canada)**[2]	Quasi-experimental design using clinical sample	Clinical sample (inpatient and outpatients) classified by DSM-III-R (1987) criteria. AN-Restrictor N =26; AN with bulimia = N = 16. EDNOS with low body weight N = 11. Mean AN BMI = 16.5. Classified as high level exercisers (N = 22, mean age = 27.1) or moderate/non exercisers (N = 31, mean age = 28.8).	Explored relationships between exercise levels, Obsessive Compulsive symptomatology, and restricted eating in AN. Discussed in relation to models of biological mechanisms in AN.	Questionnaires completed, physical activity interview for exercise classification. Height and weight measured after interview.
**Davis et al. (1995) (Canada)**[39]	Cross-sectional study using clinical and non-clinical sample	Clinical sample; Inpatients with AN, or had met criteria within past year for AN (N = 46, Mean age = 24.2, SD = 4.7). Non-clinical samples: 2 samples of women (n = 88, Mean age = 23.3, SD = 3.8 and n = 40, Mean age =24.7, SD = 3.2)	Aimed to investigate relationship between obsessive-compulsiveness and psychological and behavioural aspects of exercise in women with AN	Clinical sample tested within first 5 or 6 weeks of hospital admission, through questionnaires and interview.
**Holtkamp et al. (2004) (Germany)**[40]	Cross-sectional study using clinical sample	30 female adolescent inpatients with AN. Mean age = 14.6 years. Mean BMI = 14.4 kg/m^2^.	Examined relationships between restricted diet, increased physical activity and psychopathology in acute stage of AN.	All patients diagnosed by experienced clinician, AN subtype diagnosed by trained interviewer blind to study hypothesis within three days of admission.
**Naylor et al. (2011) (UK)**[41]	Cross-sectional study comparing clinical and non-clinical samples	Clinical sample recruited from 4 eating disorder services: AN =30; BN = 24; EDNOS = 10. Mean BMI = 19.23, Mean age =29.98. Non-clinical university student sample: mean BMI = 20.86, Mean age = 20.32.	Aimed to explore exercise beliefs, obsessive beliefs and obsessive compulsive behaviours to understand the role of excessive exercise in eating disorders.	Clinical participants assessed and diagnosed by experienced clinicians using semi-structured interview, permission granted to gather diagnoses, BMI from medical file. Nonclinical sample reported height and weight.
**Penas-Lledo et al. (2002) (Spain)**[31]	Cross-sectional design using retrospective case notes from inpatient service	ANR patients = 35; ANBP patients = 28. BN patients = 61. Mean age = 20.25 years. Mean AN BMI = 16.9	Examined whether physical exercise is related to different aspects of psychopathology and if this association is different between diagnoses.	Data collected routinely by clinicians blind to the hypotheses. Participants coded as excessive exercisers if exercised at least 5× week (>1 hr per session), with aim to burn up calories.
**Shroff et al. (2006)**[42]**USA**	Cross-sectional design using clinical sample, and relatives with history of eating disorder.	AN probands and biological relatives who met lifetime diagnosis of AN, N = 431. BN probands and biological affected relatives, N = 750. AN Trios study- probands and parents, N = 749. Resulting sample size = 1857.	Explored features associated with excessive exercise across subtypes of eating disorders.	Participants from multi-site international Price Foundation Genetic study. Probands and relatives assessed for psychological and personality features that may underlie vulnerability to eating disorders.

**Table 3:** Outcome measures and results of included studies

***Authors (Year) (Country)***	***Outcomes***	***Quality index assessment***	***Results relating to OCD***	***Results relating to OCPD***	***Limitations***
**Anderluh et al. (2009) (Slovenia/UK)**[35]	EATATE^1^ to assess diagnosis, screen for lifetime obsessive compulsive disorder and eating disorder diagnosis using ICD-10 criteria; as well as obsessive compulsive traits in childhood.	9	Groups did not differ in lifetime duration of excessive exercising. No differences between groups in frequency of current or lifetime OCD.	No differences between groups in frequency of current OCPD. Children who were rule bound/cautious exercised excessively later in life (*p* < .005, *p < .*02).	Retrospective assessment subject to memory biases, although anchor points were used. Data from informants could have assisted with this. Participants recruited from secondary and tertiary treatment centre.
**Bewell-Weiss & Carter (2010) (Canada)**[36]	EDE^2^; EDE-Q; BSI for anxiety; BDI-II for depression; RSES for self-esteem; Padua Inventory for obsessive compulsive symptoms; EDI for eating disorder attitudes and behaviours.	11	Overall model significant (*p* < .05) explained 31% of variance in exercise status. Restraint, self esteem and depression positively associated with exercise (*p* < .05), OC symptomatology negatively associated with exercise status (*p = .*038).	NA	Need to replicate findings with other measures. Study of factors associated with specific definition of exercise. Cross-sectional nature could not demonstrate direction of associations. No measure of OCPD.
**Davis & Claridge (1998) (Canada/UK)**[37]	EPQR^3^ for addictiveness; obsessive-compulsive personality subscales; Drive for thinness for weight preoccupation; Interview to determine lifetime and current exercise status- classified as excessive or non-excessive exercisers.	11	NA	Both addictiveness and obsessive-compulsiveness were positively associated with over-exercising (both currently and historically, *p* < .05 and *p* < .01 respectively).	Patients were specifically chosen to represent the two diagnoses, although commonly both AN and BN features co-occur in clinical syndromes and in personality structure of patients.
**Davis & Kaptein (2006) (Canada)**[38]	Interview to determine lifetime and current exercise status (excessive or non-excessive); MOCI^4^ to assess for OCD symptomatology; obsessive-compulsive personality subscales; BMI.	11	Excessive exercisers showed higher intensity/number of OCD symptoms than non-excessive patients (*p* = .007). There was a decline in symptom severity between admission and discharge *(p < .*001).	Excessive exercisers demonstrated greater OC personality traits than non-excessive patients (*p = .*03). There was no significant decline in OC personality traits between admission and discharge.	Self-report recall data was used in this study. Indirect historical data is necessary, on account of low prevalence of AN-R. Difficulties with prospective designs.
**Davis et al. (1998) (Canada)**[2]	MOCI^5^ -symptoms of OCD; Obsessive Compulsive Personality subscales; MPS for perfectionism; CES for commitment to exercise; EDI: weight preoccupation; BES for body image; JFFIS for self-esteem; BMI.	11	Exercisers scored significantly higher than non-exercisers on OC symptomatology, (*p* = .02). Exercisers also reported more obligatory and pathological attitudes to exercise (*p* < 0.01).	Exercisers scored significantly higher than non-exercisers on OC personality characteristics (*p < .*05), and self-oriented perfectionism (*p < .*05).	No information as to whether exercise and obsessionality influence prognosis. Obsessionality data obtained solely from self-report data, not structured diagnostic interview.
**Davis et al. (1995) (Canada)**[39]	SCL-90^6^ to measure obsessive-compulsiveness; Drive for thinness to measure weight preoccupation; CES to measure commitment to exercise; interview to assess for physical activity.	12	Obsessive-compulsiveness significantly positively related to level of activity among AN patients (*p* < .01), and obligatory and pathological aspects of exercise were related to weight preoccupation (*p* < .01).	NA	Proposed activity-based anorexia model explains AN development only for some individuals. Does not take into account motivational factors, differences in selecting forms of exercise and reasons for exercising. No OCPD measure.
**Holtkamp et al. (2004) (Germany)**[40]	SIAB^7^ to assess AN subtype; SCL-90-R to assess for anxiety, depression and obsessive-compulsiveness.	11	Obsessive-compulsiveness was not associated with physical activity levels (*r* = -.072, *p* = .705). Regression model based on BMI, food restriction, subtype, anxiety, depression and obsessive-compulsiveness (OC) explained 64% of variance in model, OC was not a significant contributor.	NA	Small sample size; data on food restriction were answered retrospectively; need more detailed measure of OCD symptoms; need to examine OCPD symptoms; SCL-90-R only validated for people 14 years and older.
**Naylor et al. (2011) (UK)**[41]	Exercise Frequency; CET^8^ to measure beliefs about exercise; OBQ-44 to assess OCD constructs; OCI-R to assess distress associated with OCD symptoms; EDE-Q.	12	Clinical: women with higher exercise beliefs had higher levels of obsessive beliefs and obsessive compulsive behaviours (*p* < 0.01). OBQ and OCI-R accounted for significant increase in variance of weight control exercise explained.	NA	Cross-sectional design of study does not show direction of variables; rather just associations. Use of Self-report measures risks of socially desirable responses. Student samples are arguably unrepresentative of general population, could use other control groups.
**Penas-Lledo et al. (2002) (Spain)**[31]	EAT-40^9^: overall level of eating pathology. BITE: bulimic attitudes and behaviours. SCL-90-R: current psychological symptoms BMI.	11	AN patients who exercised had higher levels of eating pathology (EAT; *p* < .01) Exercisers had higher levels of anxiety and depression on SCL-90-R (*p* < .01), but not OCD symptoms (*p* > .05).	NA	Problems in definition of excessive exercise used to classify groups; require different measures to examine OCD symptoms in more detail; no measurement of OCPD symptoms
**Shroff et al. (2006)** (**USA)**[42]	Clinical variables: ED duration, current/minimum/maximum BMI obtained; SIAB; SCID for diagnosis; TCI for temperament; MPS for perfectionism; STAI for anxiety; Y-BOCS for OC symptoms; YBC-EDS; excessive exercise classification from SIAB.	13	Excessive exercise was associated with greater severity of ED symptoms, worst ritual, preoccupation and worst motivation to change in YBC-EDS. Also associated with higher obsessions and compulsions (YBOCS) (*p < .*001)	Excessive exercise was associated with all perfectionist traits (p < .001), as measured by MPS.	Exercise group determined by retrospective reports of exercise behaviour. Exercise assessment not comprehensive. Unable to determine association between duration of excessive exercise and other ED behaviours.

^1^*EATATE* EATATE Lifetime Diagnostic Interview [43].

^2^*EDE* Eating Disorder Examination [44]; *EDE-Q* Eating Disorder Examination-Questionnaire [45]; *BSI* Brief Symptom Inventory [46]; *BDI-II* Beck Depression Inventory [47].

*RSES* Rosenberg Self-Esteem Scale [48] Padua [49]*EDI* Eating Disorder Inventory [50].

^3^Eysenck Personality Questionnaire-Revised [51];Obsessive-compulsive personality subscales [52]; Subscale from the Eating Disorder Inventory [50]; exercise interview as used previously by [21,30].

^4^*MOCI* Maudsley Obsessive-Compulsive Inventory [53]; personality subscales [52].

^5^*MOCI* Maudsley Obsessive-Compulsive Inventory [53]; personality subscales [52]; *MPS* Multidimensional Perfectionism Scale [54]; *CES* Commitment to Exercise Scale [55]; *EDI* Eating Disorder Inventory [50]; *BES* Body Esteem Scale [56]; *JFIS* Janis-Field Feelings of Inadequacy Scale [57].

^6^*SCL-90* Symptom Checklist-90 [58]; *Drive for thinness* from EDI [50]; CES [55].

^7^*SIAB* Structured Interview of Anorexia and Bulimia Nervosa [59]; SCL-90-R [60].

^8^*CET* Compulsive Exercise Test [32]; *OBQ-44* Obsessive Beliefs Questionnaire-44 [61]*OCI-R* Obsessive Compulsive Inventory-Revised [62]; *EDE-Q* Eating Disorder Examination-Questionnaire [63].

^9^*EAT-40* Eating Attitudes Test [64]; *BITE* Bulimic Investigatory Test [65]; SCL-90-R [66]; *SIAB* Structured Interview for Anorexia and Bulimia Nervosa [59]; SCID [67]; *TCI* Temperament and Character Inventory [68]; Frost MPS [54]; STAI [69] Y-BOCS [70] YBC-EDS [71].

### Quality assessment

The total mean score on the Quality Index was 11.2/15 and scores ranged from 9–13. Refer to Table 1 for the quality assessment of each study. The mean subscale scores were 5.9/7.0 (range 5–7) for reporting, 1.2/3.0 (range 0–2) for external validity and 4.0/4.0 for internal validity. One study reported a power calculation.

### Measures used

Objective measures of height and weight were collected in all studies. Structured interview schedules for exercise behaviours included different versions of the Eating Disorder Examination (EDE) interview [44]. These structured interviews assessed the exercise behavior of the participant over the past three months, asking about exercise that was “obligatory” or “obsessive” or “driven”, and engaged in for the purpose of burning calories/kilojoules or weight control. The Structured Interview for Anorexic and Bulimic Disorders (SIAB) from DSM-IV and ICD-10 [59] was used in other studies to separate excessive from non-excessive exercisers, through the endorsement of any of the following categories of exercise behaviour: 1) severe interference with important activities; 2) exercising more than 3 hours/day and distress if unable to exercise; 3) frequent exercise at inappropriate times and places; and 4) exercising despite illness, injury or medical complications. Other measures were questionnaires including the Commitment to Exercise Scale (CES: [55]) which assesses the obligatory pathological aspects of exercise and the Compulsive Exercise Test (CET: [32]) which assesses avoidance and rule-driven behavior, weight control exercise, lack of exercise enjoyment, mood improvement, guilt, negative and positive reinforcement of exercise, and behavioural rigidity.

Other assessment protocol for excessive exercise included questions regarding duration, frequency and intensity of exercise per week [2,21,30] and questions of lifetime exercise status.

OCD symptomatology was measured using different self-report questionnaires. The OBQ-44 assessed constructs of inflated responsibility/threat estimation, perfectionism/tolerance of uncertainty, and importance/control of thoughts [61]. The Obsessive Compulsive Inventory-Revised [62] was utilised to assess the distress associated with symptoms of OCD, such as checking, washing, obsessing and ordering. The Padua Inventory [49], Yale-Brown Obsessive Compulsive Scale [70] and the Maudsley Obsessive-Compulsive Inventory [53] measured similar constructs to the OCI-R. A number of other studies used the Symptom Checklist-90-R [66] to measure obsessive-compulsive symptoms.

OCPD traits were measured through a number of methods. The EATATE interview [43] was included in one study to assess for obsessive-compulsive traits in childhood (such as perfectionism, drive for order and symmetry, and excessive doubt). Two other studies used an inventory designed to assess the “obsessional” personality type derived from psychoanalytic theory as a measure of obsessive-compulsive personality traits [52], and two studies used the Multidimensional Perfectionism Scale [54] to measure perfectionism, demonstrated to be one of the main temperamental characteristics of AN [72].

### Relationship between excessive exercise and OCD symptomatology

Davis and Kaptein [38] demonstrated that patients who were identified as excessive exercisers showed higher number of obsessive-compulsive symptoms (*p* = .007) than non-excessive exercisers, unaffected by dietary status. Both excessive exercisers and non-excessive exercisers demonstrated a reduction in OCD symptoms between admission and discharge (*p < .*001). There was an interaction trend demonstrating that, after re-feeding, OC symptoms decreased less in the excessive exercisers group. It was also noted that patients who presented with excessive exercise reported a higher number of obsessive compulsive symptoms on the Maudsley Obsessive Compulsive Inventory (at admission and discharge) than a group of patients diagnosed with OCD [73].

Similar findings were demonstrated in studies by Davis et al. [2,39] in which AN patients who exercised excessively reported more obligatory and pathological attitudes towards exercise (*p* < .01). Obsessive-compulsiveness was positively related to level of activity in AN patients (*p < .*01) and exercising participants also demonstrated higher OC symptomatology than non-exercisers (*p* = .02).

Naylor et al. [41] concluded that women with AN who had higher levels of beliefs of exercise behaviour also had higher levels of obsessive beliefs (*p* < .01), obsessive-compulsive behaviours (*p* < .01), as well as higher eating disorder psychopathology (*p* < .01). Specifically, the Checking subscale from the OCI-R contributed uniquely and significantly to the overall model explaining weight control exercise, signifying that these obsessive beliefs and behaviours predict the variance in exercise for purpose of weight control, after controlling for eating disorder psychopathology. Furthermore, Shroff et al. [42] reported that excessive exercise was associated with higher frequency and intensity of rituals and preoccupations (*p* < .001), and higher frequency of obsessions and compulsions (*p* < .001), when compared with AN patients who completed no or regular exercise.

However, Anderluh et al. [35] found no differences between groups in frequency of current or lifetime OCD. Bewell-Weiss & Carter [36] demonstrated that although self-esteem and depressive symptomatology were positively associated with exercise, obsessive compulsive symptomatology was negatively associated with exercise status (*p* = .038). Penas-Lledo et al. [31] found that OCD symptoms were not significantly higher in patients who exercised excessively when compared with those who did not (*p* > .05). Finally, Holtkamp et al. [40] concluded that obsessive-compulsiveness was not associated with physical activity levels (*p* = .705) and that obsessive-compulsiveness was not a significant contributor in the regression model predicting physical activity with other factors such as BMI, level of food restriction, depression and anxiety.

### Relationship between excessive exercise and OCPD traits

Davis and Kaptein [38] demonstrated that patients who were excessive exercisers showed a higher number of obsessive-compulsive personality traits, both currently and historically throughout their disorder (*p* = .03) than non excessive exercisers, and this was unaffected by dietary status.

Davis et al. [2] showed that excessive exercisers had higher OC personality characteristics (*p* < .05) and levels of self-oriented perfectionism (*p < .*05) than non-excessive exercisers. Anderluh et al. [35] reported that patients with AN who exercised excessively had a higher prevalence of OCPD traits during childhood such as being rule bound (*p <* .005) and cautious (*p < .*02), however they did not find any significant differences in current OCPD comorbidity (*p* > .05).

Davis and Claridge [37] demonstrated that obsessive-compulsive personality traits were positively associated with over-exercising, both in current excessive exercising (*p* < .05) and historically throughout the eating disorder (*p* < .01). Furthermore, Shroff et al. [42] reported that excessive exercise was associated with significantly greater perfectionism (*p* < .001), measuring factors such as concern over mistakes, personal standards, organisation and parental criticism.

## Discussion

The aims of this systematic review were to critically examine evidence as to whether OCPD traits and/or OCD are associated with excessive exercise in patients with AN, and to determine the nature of relationships between these constructs. The results of the systematic review indicated a positive relationship between excessive exercise and obsessive-compulsive personality traits. However, the relationship between OCD and excessive exercise in AN patients is less clear with studies producing varying results.

Davis et al. [39] proposed a theoretical model of the relationship between starvation, physical activity and obsessive-compulsiveness in the development of eating disorders for some patients. This model works on the understanding that significantly reduced dietary intake and increased physical activity, combined with OCD features, create a mutually reinforcing, destructive mechanism which may play an integral role in the development and maintenance of an eating disorder [39]. The results of this review also seem consistent with the theory of “activity anorexia” [23], providing preliminary evidence for this phenomenon in human samples. Results have also supported the notion that high level exercising and reduced dietary intake alter the functioning of 5-HT with increased OCD symptomatology [2], creating a cycle whereby the individual undertakes an even higher level of physical activity and decreases their dietary intake as their obsessions increase [39].

Furthermore, the findings of Naylor et al. [41] are consistent with research examining the reduction in quality of life for these patients [74] and descriptions of their exercise being “out of control” [39]. Having obsessive beliefs and compulsions was a significant predictor in the regression model for exercise beliefs, after controlling for BMI and eating disorder psychopathology [41]. Such findings are consistent with the premise that obsessionality plays a causal role in the development and maintenance of excessive exercise [38].

Bewell-Weiss and Carter [36] reported a negative relationship between OCD symptomatology and excessive exercise. The researchers concluded that it may have been that their regression model was more comprehensive than those used in previous studies, or that obsessive-compulsive symptomatology had shared variance with another variable that had not been explored with previous studies [36]. Additionally, they used different OCD measures than those used in other studies [36]. Penas-Lledo et al. [31] found a trend only for increased obsessive compulsive symptomatology in exercising patients using the SCL-90-R. Finally, Hotlkamp et al. [40] found no direct relationship between obsessive-compulsiveness and levels of physical activity, also assessed using the SCL-90-R. It may be that this measure is not as sensitive as other measures, or that OCD features affect cognitions or beliefs about exercise [40].

Davis et al. [2] speculated that it could be that greater obsessive-compulsive personality traits are exacerbated by the combination of starvation and high level exercise, or that these patients choose to combine dietary restraint with excessive exercise. It may be the case that patients who have obsessive tendencies are more likely to undertake exercise as an additional method to prevent weight gain (on top of restricted diet, purging behaviour) or to neutralise fears of changes in their body weight and shape [75]. Alternatively, patients may be using exercise to alleviate or reduce anxiety [76], or as a means of neutralising predominant weight or food related obsessions [40].

Anderluh et al. [35] found that participants with childhood traits of rigidity, extreme cautiousness and perfectionism underwent more severe food restriction and higher levels of excessive exercise, and experienced longer periods of underweight status. Their research identified the possibility of homogenous phenotypes of AN and demonstrates that premorbid obsessive-compulsive personality traits in childhood may influence the course of the eating disorder later in life, potentially contributing to a more severe form of restricting AN, which could be extremely resistant to treatment [38].

There are a number of limitations that were evident in the reviewed literature. The number of studies examined in the review (10) is small, and results must be interpreted with some caution as some of the studies did not support the association between OCD and excessive exercise. A number of the reviewed studies did not clearly differentiate between OCD and OCPD constructs in their presentation of results. Nine different types of measures were employed across the studies to assess OCPD traits and OCD symptomatology, many of these being self-report questionnaires, which may increase the incidence of socially desirable response styles. Others involved expert clinical assessment in the form of an interview (for example, the EATATE interview in Anderluh et al. [35]). These various instruments assess different constructs, and some measures may have provided more detailed information, both of which may have biased the results. In one study, the use of the SCL-90 may have been inappropriate, as it had been normed for participants aged 14 years and older, yet a proportion of their sample were aged between 13 and 14 years. It would thus be beneficial for future studies to utilise the most current and comprehensive assessment tools in the field to enable more reliable inter-study comparisons.

Denial commonly occurs in anorexia nervosa, and subjective measures may have underestimated the amount of physical activity completed by patients. Such underestimation may have led to significant bias and consequently inaccuracy of data regarding the extent of exercise, and its relationships with coexisting OCD and/or OCPD. This potential room for bias has led to the utilisation of more objective means of assessing for physical activity in patients with AN, for example accelerometers [77,78]. Yet no study included in the review used such devices. Numerous measures of exercise were utilised across studies, reflecting both the variety of measures available, and the time period over which the studies were undertaken (1995–2011). However, there is also a pervasive issue in this field regarding the lack of consensus on what defines excessive exercise, how it should be measured and how it should be managed in patients with AN [79].

There are also a number of limitations in regards to participants utilised in the studies. The small sample size of Holtkamp et al. [40] affected their capability to generalise their results, whilst the use of informants may have been beneficial for Anderluh et al. [35] to confirm retrospective data provided by participants and to limit memory bias effects. Participants across studies who were inpatients were not exercising at the time of completing study assessments and questionnaires, and it is unknown what effect this may have had on response style. Other participants (for example outpatients) would not have such intense restrictions. The research reviewed also did not take into account other factors which may be important in the study of obsessive-compulsive symptomatology with excessive exercisers, such as motivational factors and specific reasons for exercising (other than the use of CET in Naylor et al., [41]). Finally, across studies, the patients were recruited mostly from secondary and tertiary referral services, i.e. inpatient and outpatient eating disorder services. Thus, the findings from the review cannot be generalised to people with AN who are currently not seeking treatment, or whose eating disorder pathology is not as severe as those patients in hospital treatment.

As the vast majority of the studies employed a cross-sectional design, only relationships between variables could be determined, and there can be no demonstration of the direction of such associations. Acute starvation syndrome and severity of eating disorder psychopathology both have significant impact upon level of obsessive-compulsive symptoms and exercise [17]. There was also no information about how obsessionality might affect prognosis and treatment outcome for AN patients who exercise excessively.

Whilst this review has focused on the relationships between OCPD traits and/or OCD symptomatology with excessive exercise in patients with AN, it would be remiss not to mention the significant relationships which excessive exercise shares with other psychopathologies, which have important implications for treatment. Studies included in the review demonstrated that AN patients who exercised excessively demonstrated lower minimum BMI and lower novelty seeking, but higher harm avoidance, persistence and cooperativeness [42]. In other studies, these patients demonstrated higher levels of anxiety and food restriction [40]; higher levels of depression, self-esteem and dietary restraint [36]; higher addictive personality traits [37]; higher weight preoccupation [39]; and higher levels of bulimic and eating disorder psychopathology [31].

## Conclusions

From the research reviewed, it appears that there is a positive association between obsessive- compulsive personality traits in patients with AN who excessively exercise, yet the relationship with obsessive-compulsive disorder is less clear. Although it is known that excessive exercise is associated with poor treatment outcome in AN [28,29] the effects of obsessionality on treatment outcome are not yet known. Results of this paper indicate that obsessive-compulsive symptomatology and/or obsessive-compulsive personality traits contribute greater complexity to individual cases and may make these patients more resistant to treatment. Results support integrated interventions addressing the role of driven exercise in the context of obsessionality and obsessive compulsive symptoms where present, providing therapeutic interventions to alleviate emotional distress [31] and addressing characteristics such as perfectionism and behavioural rigidity [80]. The Loughborough Eating Disorders Activity Programme (LEAP) is an exemplar in this regard [81]. It is a manualised intervention which addresses driven exercise behaviours and beliefs using psycho-education and specific cognitive behavioural techniques to manage unhelpful beliefs and attitudes towards exercise, negative affective and compulsivity. Finally, future research into the relationship between driven exercise and obsessionality should utilise the most comprehensive and reliable clinical assessment tools, and address prognostic factors, treatment factors and specific interventions for patients with OCPD and/or OCD and driven exercise.

## Competing interests

The authors declare that they have no competing interests.

## Authors’ contributions

SY undertook the systematic review and with PH prepared this manuscript for journal submission. PH, ST and PR assisted with the editing of the manuscript and data interpretation. All authors read and approved the final manuscript.

## Authors’ information

SY submitted an alternate version of this manuscript as a requirement for the Doctor of Clinical Psychology/Master of Science thesis at University of Sydney. She is supervised by ST, PH and PR. PH, ST and PR are conducting a randomised controlled trial and evaluation of the Loughborough Eating Disorders Activity Programme (LEAP) referenced in this paper.
